# Pericardial Cyst at an Unusual Location: The Role of CT Imaging

**DOI:** 10.7759/cureus.42403

**Published:** 2023-07-24

**Authors:** Pokhraj P Suthar, Avin Kounsal, Lavanya Chhetri, Chetankumar M Mehta, Shehbaz M Ansari, Raman Shingade

**Affiliations:** 1 Department of Diagnostic Radiology and Nuclear Medicine, Rush University Medical Center, Chicago, USA; 2 Department of Clinical Nutrition, Rush University Medical Center, Chicago, USA; 3 Department of Radiodiagnosis, Sir Sayaji General Hospital & Medical College Baroda, Vadodara, IND; 4 Radiology, Nidan Diagnostic and Research Center, Bhubaneswar, IND

**Keywords:** chest pain, ct, pericardial cyst, mediastinum, chest

## Abstract

The pericardial cyst is a benign, uncommon congenital cystic lesion of pericardial origin located in the anterior and middle mediastinum. Most commonly, pericardial cysts are located in the right anterior cardiophrenic angle. Computed tomography (CT) and magnetic resonance imaging (MRI) of the chest are the non-invasive imaging modalities for the diagnosis of the pericardial cyst. Here we present a case of a 55-year-old male who presented with coughing and chest pain for two weeks. A chest X-ray revealed a soft-tissue opacity mass in the left lower zone. A CT of the chest showed a fluid-density cystic lesion in close proximity to the pericardium, located along the left posterior cardiophrenic angle, an uncommon location for a pericardial cyst.

## Introduction

Pericardial cysts are rare benign mediastinal abnormalities [1]. They are usually congenital and occur as a result of the abnormal formation of coelomic (somatic) cavities. Some cases may be acquired, usually after cardiothoracic surgery. The cyst wall contains connective tissue and mesothelial cells. They are most commonly found on the right, in particular the right anterior cardiophrenic angle. The majority of the patients are asymptomatic and have an incidental diagnosis, while CT or MRI are performed for other etiologies. The patient develops symptoms in a large pericardial cyst secondary to the compression of adjacent organs [2]. A diagnosis may be suspected in cases with abnormal chest X-rays showing an abnormal opacity adjacent to the cardiac border. CT scans and MRIs of the chest are the non-invasive imaging modality of choice for the accurate diagnosis and characterization of pericardial cysts and to differentiate them from other cystic mediastinal masses [3]. An asymptomatic small-size pericaridal cyst requires closed follow-up for an increase in size [4]. Large pericardial cysts or cysts with clinical symptoms should be surgically aspirated or excised [5].

## Case presentation

**Figure 1:**
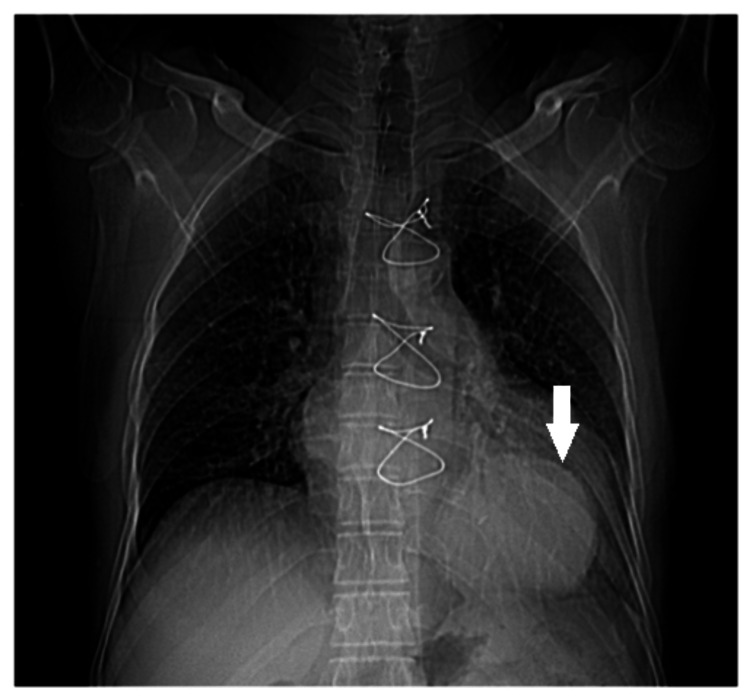
Scout film showing a well-defined, round density in the left lower zone overlapping the heart shadow adjacent to the left cardiophrenic angle (white arrow)

**Figure 2:**
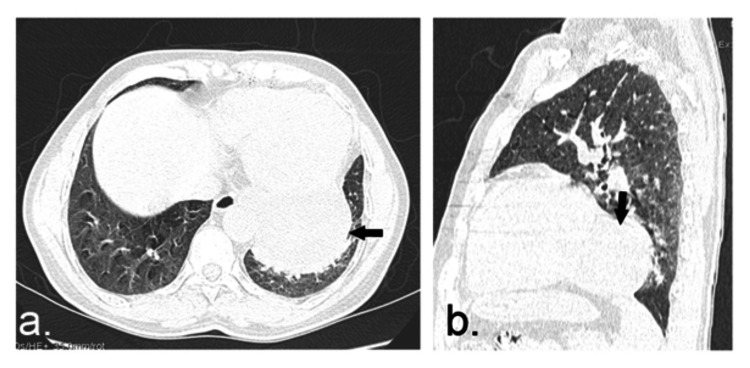
(a) axial and (b) sagittal unenhanced CT (lung window) images show abnormal, round-oval lesion (black arrows) along the left posterior cardiophrenic angle

**Figure 3:**
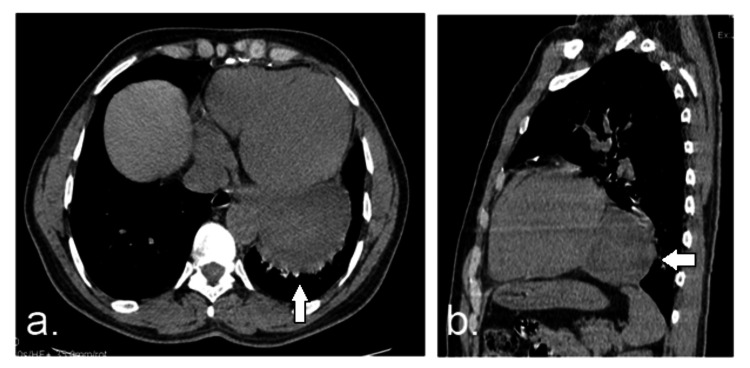
(a) axial and (b) sagittal unenhanced CT (mediastinal window) images show the fluid density lesion located in the left posterior cardiopheric angle in close proximity to the pericardium (white arrow)

**Figure 4:**
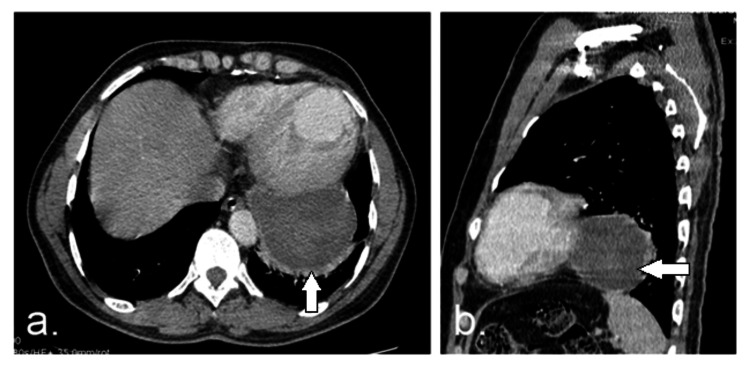
(a) axial and (b) sagittal contrast-enhanced CT (mediastinal window) images show non-enhanced fluid density lesions located in the left posterior cardiopheric angle in close proximity to the pericardium (white arrow) with associated compressive atelectasis of adjacent pulmonary segments

A 55-year-old male, non-smoker, presented with complaints of a non-productive cough and chest pain for two weeks. The patient denied any palpitations, syncopal episodes, paroxysmal nocturnal dyspnea, orthopnea. There was no history of deep vein thrombosis or embolism. The patient had a prior median sternotomy for open heart surgery, but prior clinical details, X-rays, or documents were not available. The patient denied any significant past medical or family history. The review of systems was negative; he denied any history of recent travel, trauma, camping, or vaccination. The patient was not taking any dietary supplements, nor was he taking prescription medication. Examination revealed vital signs were within normal limits (temperature 97.3 °F (36.3 °C), heart rate 80 beats per minute, SpO_2_ 100%, respiratory rate 16 per minute, and blood pressure 116/88 mm Hg). The systemic examination, including neurological, respiratory, cardiovascular, and abdominal, was within normal limits. Routine blood investigations, including the blood counts, urea, and electrolytes, were all within normal limits. The cardiac enzymes were not elevated. An electrocardiogram (EKG) was unremarkable. A chest X-ray revealed an abnormal radiopacity in the left lower zone, posterior to the heart. A CT of the chest with contrast was advised. Scout film showed cardiomegaly with a well-defined, round density in the left lower zone, posterior to the heart, and adjacent to the left cardiophrenic angle (Figure 1). An unenhanced CT scan (lung window) showed abnormal, round-oval hypodensity along the left posterior cardiophrenic angle (Figure 2). On mediastinal window images, the lesion was seen to be of fluid density and located in close proximity to the pericardium (Figure 3). Contrast-enhanced images showed non-enhancing lesions with associated compressive atelectasis of adjacent pulmonary segments (Figure 4). CT-guided percutaneous aspiration was performed, which yielded an exudative fluid. Considering the prior history of cardiac surgery and the exudative nature of the pericardial fluid, the diagnosis of a pericardial cyst secondary to the prior surgery was postulated. The patient underwent thoracoscopic excision of the lesion, and the patient was discharged after two weeks with complete relief of symptoms. On follow-up after one month, the patient had no new complaints.

## Discussion

A pericardial cyst, also called a mesothelial cyst of the pericardium, is a rare condition with an incidence of 1 in 100,000 [6]. They are usually unilocular and contain a clear, water-like fluid. The cysts can be of variable size, ranging from 2-28 cm [7]. Pericardial cysts are usually asymptomatic, but in some cases, symptoms may occur because of compression of adjacent structures [2]. In symptomatic cases, patients may present with chest pain, cough, fever, and arrhythmias. Rarely, it may result in serious complications like erosion into vascular structures, pericarditis, obstruction of the right ventricular outflow tract, pulmonary stenosis, or sudden death [8]. 

The most common location for the pericardial cyst is the right anterior cardiophrenic angle, with the second most common location being the left anterior cardiophrenic angle. However, in occasional cases, a pericardial cyst may arise elsewhere in the mediastinum [8].

Most often, a pericardial cyst is an incidental finding on chest radiography or echocardiography. On radiography, these are well-defined, round or oval masses in the cardiophrenic angle. Ultrasonography can reveal the cystic characteristics of the lesion. However, the shape of the lesions is not always round and can vary greatly when imaged at different times [9].

For the diagnosis of pericardial cysts, Hynes et al. first used two-dimensional (2D) echocardiography [1]. 2D echocardiography with color duplex is a non-invasive imaging modality for the diagnosis of the pericardial cyst and helps to determine that the mass is fluid-filled and to rule out potential vascular differentials including left ventricular aneurysm, aortic aneurysm, or prominent left atrial appendage. Transesophageal echocardiography is an invasive modality but useful for the diagnosis of atypical locations of pericardial cysts [2].

On CT, these are well-defined, fluid-density lesions in close proximity to the pericardium. However, at surgery, very few cases demonstrate definite communication with the pericardial sac [9]. The lesions do not enhance on post-contrast images and contain no septation within. The lesion may show intermediate attenuation on CT if the cyst fluid is proteinaceous. Also, hemorrhagic cysts may show hyperattenuation on CT.

On MRI, pericardial cysts will show intermediate to low signal intensity on T1-weighted images and high signal intensity on T2-weighted images. In the case of proteinaceous contents within the cyst, high signal intensity on T1-weighted sequences and variable intensity on T2-weighted sequences may be noted. Pericardial cysts show no enhancement in a T1 post-contrast (Gd) sequence.

Acquired causes of the pericardial cysts include inflammation from prior cardiac surgery, trauma, tuberculosis, echinococcosis, pericarditis, metastasis, or hemodialysis. It's difficult to differentiate congenital pericardial cysts from cysts secondary to surgery on imaging, but CT scan and MRI show an encapsulated fluid-filled structure without internal septa or hemorrhage, favoring congenital pericardial cysts [10].

The majority of pericardial cysts usually have a benign course, although potential reported complications include erosion of the cyst into the right ventricular wall [11] or superior vena cava [12], cyst rupture [2], cardiac tamponade [13], obstruction of the right main bronchus [14], mitral valve prolapse [15], or sudden death [16].

Asymptomatic small-size pericardial cysts require closed follow-up for an increase in size [4]. A few cases managed conservatively reported spontaneous resolution [15, 17]. Management involves surgical intervention and is usually reserved for symptomatic patients or in the presence of complications. Either surgical resection or percutaneous drainage can be performed [5].

## Conclusions

Pericardial cysts are rare, benign conditions that may be congenital or acquired. The most common location for the pericardial cyst is the right anterior cardiophrenic angle, followed by the left anterior cardiophrenic angle. A CT scan aids in the early and accurate diagnosis and characterization of pericardial cysts. Here we present the case of an unusual location of the pericardial cyst located in the left cardiophrenic angle, which raises the reader's awareness.
